# Multimodal Communication in a Noisy Environment: A Case Study of the Bornean Rock Frog *Staurois parvus*

**DOI:** 10.1371/journal.pone.0037965

**Published:** 2012-05-24

**Authors:** T. Ulmar Grafe, Doris Preininger, Marc Sztatecsny, Rosli Kasah, J. Maximilian Dehling, Sebastian Proksch, Walter Hödl

**Affiliations:** 1 Department of Biology, Universiti Brunei Darussalam, Gadong, Brunei Darussalam; 2 Department of Evolutionary Biology, University of Vienna, Vienna, Austria; 3 Department of Animal Ecology and Tropical Biology, University of Würzburg, Theodor-Boveri-Institut, Biozentrum, Würzburg, Germany; The Australian National University, Australia

## Abstract

High background noise is an impediment to signal detection and perception. We report the use of multiple solutions to improve signal perception in the acoustic and visual modality by the Bornean rock frog, *Staurois parvus*. We discovered that vocal communication was not impaired by continuous abiotic background noise characterised by fast-flowing water. Males modified amplitude, pitch, repetition rate and duration of notes within their advertisement call. The difference in sound pressure between advertisement calls and background noise at the call dominant frequency of 5578 Hz was 8 dB, a difference sufficient for receiver detection. In addition, males used several visual signals to communicate with conspecifics with foot flagging and foot flashing being the most common and conspicuous visual displays, followed by arm waving, upright posture, crouching, and an open-mouth display. We used acoustic playback experiments to test the efficacy-based alerting signal hypothesis of multimodal communication. In support of the alerting hypothesis, we found that acoustic signals and foot flagging are functionally linked with advertisement calling preceding foot flagging. We conclude that *S. parvus* has solved the problem of continuous broadband low-frequency noise by both modifying its advertisement call in multiple ways and by using numerous visual signals. This is the first example of a frog using multiple acoustic and visual solutions to communicate in an environment characterised by continuous noise.

## Introduction

In any message, signals need to be successfully processed through either single or multiple channels to effectively convey information from senders to receivers [1]. Clear reception is a minimum requirement for a successful communication system [2]. Signal detectability depends on signal design, conditions of the environment, and the receiver's sensory system [2], [3]. Additional sensory stimulation in the environment can cause information to be lost. In the case of acoustic communication, noise and transmission properties of the environment may shape the spectral and temporal structure of signals [4]–[6] as well as emphasize the role of signal efficacy in the evolution of animal signals [7]. Senders can increase signal efficacy by either avoiding areas of high noise [8], overriding environmental noise [9], adjusting their signal timing [10]–[12] or by using frequencies less masked by background noise [13],[14]. Furthermore, signallers may use additional modes of communication to facilitate transmission [15],[16].

Anurans are excellent model systems to investigate acoustic communication during high levels of background noise and the advantages gained by the concomitant use of visual signals. Male advertisement calls are the principal mediators of sexual behaviour that attract females and serve to announce the readiness to defend calling sites and territories. To reduce certain patterns of acoustic interference from conspecifics and heterospecifics, individuals alter spectral or temporal call characteristics to avoid overlap [11],[17],[18], and use spatial release of masking chorus noise for species recognition [19]. Another strategy to reduce masking is to utilize multiple signal modalities, where each modality increases efficacy under specific conditions [14],[15],[20]–[22]. Visual signals may act as a complementary mode of communication in noisy habitats. For example, foot-flagging displays are conspicuous visual signals observed in tropical anuran species inhabiting fast flowing streams [15],[17],[22]–[25] or areas with heavy rains and noise produced by conspecifics [26].

Several non-mutually exclusive hypotheses have been proposed to explain the function of multimodal signals. Signals could be redundant and act independently as a back-up for increased accuracy of information transfer [27] or could contain multiple messages with each signal conveying a different message [7]. In contrast, the efficacy-based hypotheses address the factors affecting the transmission and reception of multimodal signals, with the efficacy-based alerting signal hypothesis suggesting that one signal alters the response to a subsequent signal [28]. In this study, we test the efficacy-based alerting signal hypothesis to explain the function of multimodal signals. For example, if signals of two modalities are emitted sequentially, the hypothesis predicts, among others, that the signal in one modality consistently precedes the signal of the other modality. Thus, a signal in one modality can function to alert the receiver to a subsequent signal in a different modality that might be more informative or, as is the case of visual signals, needs the receiver to look into the direction of the signaller. For example, in sticklebacks, male olfactory cues act as long distance messages that alert females to the following visual cue [29] while in the Bornean ranid frog *Staurois guttatus* vocalizations alert receivers to the subsequent foot flag [25].

In the present study, our aims are to (1) examine how the Bornean rock frog *Staurois parvus* communicates in noisy environments (2) characterize foot-flagging behaviour and other visual displays (3) record the key characteristics of their vocalizations, (4) determine the signal-to-noise ratio at a fast flowing stream in which males call, and (5) use acoustic playback experiments to test the efficacy-based alerting signal hypothesis of multimodal signalling.

## Materials and Methods

### Ethics statement

This was an observational study of free-ranging animals. The experimental protocol adhered to the Animal Behaviour Society guidelines for the use of animals in research and was approved by the Universiti Brunei Darussalam Research Committee (UBD/PNC2/2/RG/1(58)).

### Study site and species

We studied a population of *S. parvus* from 18^th^ August–26^th^ September 2005, June 2006 - January 2007 and again from 1^st^ March 2010–13^th^ April 2010 in the Ulu Temburong National Park, Brunei Darussalam, Borneo. The study site was at a narrow, rocky (black shale) section of the Sungai Mata Ikan, a small freshwater stream that merges into the Belalong River close to the Kuala Belalong Field Studies Centre (115°09′E, 4°33′N). Daily temperatures varied between 24 and 27°C. Annual precipitation at the site ranges between 2500 and 4000 mm.

**Figure 1:**
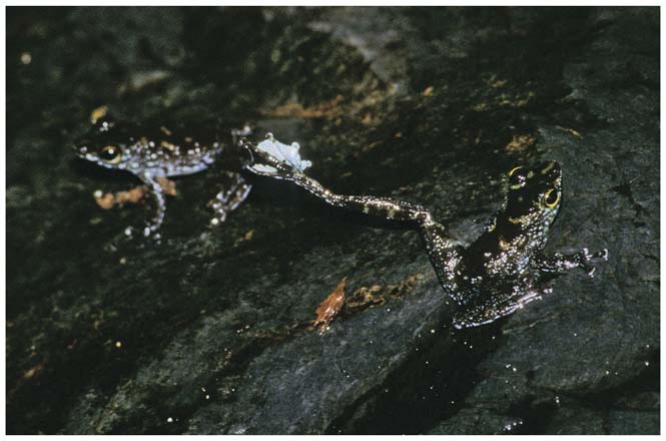
Male *Staurois parvus* foot-flagging in close proximity to a rival male.

*Staurois parvus* is a ranid frog, endemic to Borneo, recently resurrected from synonymy with *S. tuberilinguis* [30]. The separate species status has been verified using molecular markers [31]. The snout-urostyle length and weight of the investigated population of male *S. parvus* averaged 21.5±0.5 mm (SD; range 20.7–22.7; n = 13) and 0.7±0.05 g (SD; range 0.65–0.80; n = 13). Males are diurnal and perch on rocks along fast-flowing forest streams. Their white chest and white webbing between toes of hind legs strongly contrast to their cryptic dark grey, brown dorsal body (Fig. 1). Males display a conspicuous visual signal termed foot flagging during agonistic male-male encounters in which the conspicuous webbings of the hind feet are exposed [32]. Male advertisement calls have not been previously described [33].

### Behavioural observations

Behavioural sequences of acoustic and visual signals exhibited by males were recorded using continuous focal sampling [34]. Focal individual males (n = 31) were observed between 1–20 min and their activities recorded on video (Sony HC 32E PAL cam recorder; Sony Co., Japan). 40 hours of video recordings were digitized, stored on DVD and analysed.

To determine whether the vocalizations and visual foot-flagging displays function in concert or as separate entities, we determined the timing intervals between the advertisement calls and foot flags from video recordings and tested for differences using a Wilcoxon matched pairs test. Chi^2^-tests were used to test for any associations between the three most common behaviours: advertisement calls, foot flags of the left foot, and foot flags of the right foot. Further observations of signalling behaviour were recorded of male tactile behaviours and female vocal and visual signalling.

If not stated otherwise, means and SD are given as descriptive statistics and analyses were run using BIAS (v.8.2; epsilon-Verlag GbR 1989–2006). All tests are two-tailed.

### Acoustic recordings

After locating a vocalizing male, stereo recordings of the multi-note advertisement call were made from a distance of 1 m, using directional (sound left) and omni-directional microphones (Sennheiser Me 66, Me 62, Sennheiser electronic GmbH & Co. KG, Germany) and a digital recorder (Zoom HN4, Zoom Co., Japan; settings: 44.1 kHz, 16-bit resolution). Microphones were placed 50 cm apart from each other directed at the calling individual. Peak sound pressure levels (SPLs) were measured with a sound level meter (Voltkraft SL-100, Germany: settings: fast/max) during each sound recording at a distance of 1 m to the focal individual. The A-filter frequency weighting was used because it is approximately flat from 1 to 8 kHz, which comprises the call range of *S. parvus*.

Recordings with the directional microphone were used to measure call duration, note duration (each call was composed of many notes), mean-, minimum- and maximum frequency. In addition, the dependency of frequency and note duration on note number was analysed. A period of 7 s of omni-directional recordings was selected after each call to analyse the ambient noise. The sound pressure levels and energy spectra of advertisement calls and noise were compared from omni-directional microphone recordings. Furthermore, the dependency of sound pressure on note number was analysed.

The acoustic features of stereo recordings were extracted and measured using custom built programs in PRAAT 5.1.25 DSP package [35] that automatically logged these variables in an output file. To analyse single call notes the voiced intervals of the call were extracted and note duration in seconds was measured. Call duration in seconds was calculated from note start and end times. For call frequency analysis a cross-correlation algorithm was used to produce a time-varying numerical representation of the fundamental frequency (F_0_) for each call. A time step of 0.375 ms was applied over a range of 3500–6500 Hz according to the F_0_ observed on the spectrogram. From the F_0_, the parameters' mean, minimum, and maximum F_0_ in Hertz were extracted. The mean frequency value ±300 Hz was used to apply a filter before measuring sound pressure. To extract parameters from noise files, a similar analysis was applied except to measure maximum frequency of the 7 s noise file, a long-term average spectrum was computed with a bandwidth of 50 Hz. To obtain sound pressure (SP) values of ambient noise within the frequency range of the advertisement call, we applied a band-pass filter to the spectrum for frequencies from 5300–5900 Hz. The extracted relative SP values for call and noise were transformed into absolute SP (Pa) by defining the most intensive SP of the complete sound file (SP absolute = SP relative×SP measured/SP most intensive). “SP measured” corresponds to the maximum sound pressure recorded in the field.

To test the hypothesis that *S. parvus* uses frequencies that enhance the signal-to-noise ratio we compared maximum sound pressure values of ambient noise, advertisement calls and noise with a frequency filter in the range of the call frequency (labelled noise at call frequency) using Linear Mixed Models (LMMs). The statistical assumptions for LMM analysis were met (Kolmogorov-Smirnov test) and non-normal data were square-root transformed to meet the criteria. LMMs were chosen to investigate differences in sound pressure within differing number of calls per male and varying pressure values for notes per call. The sound pressure values of noise, noise filter and call, with every call consisting of 35 values for every note, were entered as a dependent variable, with the relationship of noise, noise filter and call as predictor variables. To correct for differences between male individuals, number of calls per male and number of notes per call were entered as nested random variables. For post-hoc tests we used the Student's *t* Statistic with the post-hoc sequential Bonferroni correction for alpha because of repeated pairwise comparisons.

To compare call and noise dominant frequencies the values of these parameters were entered as dependent variables with call and noise as predictor variables. A nested term was included for the identities of male (call) and call (note) as random variables to correct for differences between male individuals, number of calls per male and number of notes per call.

To test if note duration, frequency, and sound pressure are dependent on the note number of an advertisement call of *S. parvus*, the model was rerun entering either note duration, frequency, or transformed sound pressure values as dependent variables, with note number as the predictor variable. The identities of males (calls) were entered as nested random variables. All analyses were run using SPSS version 19 (SPSS Inc., Chicago, IL, USA).

### Acoustic playback experiments

To determine whether the advertisement call is used to alert other males to the subsequent visual signal, we conducted acoustic playback experiments with seven males in the field. To avoid pseudoreplication, a synthetic call based on the average call properties of five males was generated using Goldwave version 5.06 (Goldwave Inc., St. John's, Canada). Average call parameters matched those of a subsequent larger sample of calls verifying that this initial sample was representative of the population. The call consisted of 16 notes of 18 ms duration each. Each note was separated by an interval of 100 ms and had a 2-ms rise time and a 2-ms fall time. The dominant frequency of each note was set at 5770 Hz.

After suitable males were located in the field, they were presented with a five-minute silent pre-playback control prior to each five-minute advertisement call playback period. We video recorded the activities exhibited by males using a digital video cam recorder (Sony HC 32E PAL, Sony Co., Japan) set on a tripod. The playback stimulus was presented from a portable Hi-MD player (Sony MZ-RH10, Sony Co., Japan) connected to an external battery amplified speaker (SME-AFS, Saul Mineroff Electronics Inc., USA; flat ±2 dB from 100 Hz–12 kHz) placed between 40–80 cm from the focal male without disturbing it. The speaker could not be placed at a predetermined distance in the rough terrain and the distance between frog and speaker was therefore measured after the experiment to determine the sound pressure level of each playback. The sound pressure level (SPL) of the playback at a frog's position varied between 72–82 dB (re 20 µPa; Realistic sound level meter with a flat-weighted and fast-response setting). The effect of SPL on males' responses was tested using least squares linear regressions. To test for individual differences in response to the playback treatment, we used the Wilcoxon matched pairs test.

## Results

### Behavioural Displays

Male *S. parvus* showed a large repertoire of visual displays. Common displays were foot flagging and foot flashing. Less common were arm waving, upright posture, crouched posture and an open-mouth display. All displays were also seen on a regular basis outside the period of focal sampling. Males displayed from the black shale within the stream bed often immediately adjacent to running water.

Foot flagging was the most common and conspicuous dynamic visual signal produced by males (Fig. 1). It was given in both an intra- and intersexual context. Foot flags were produced by raising either the left or right hind limb off the substrate and then rotating it outward and backward in an arc during which the whitish webbing between the toes was spread and exposed. The duration of foot flags (time between the raising of the hind limb from the substrate until it is returned to the substrate) averaged 1.5±0.24 s (n = 116).

Foot flashing was similar to foot flagging, however, it lacked the phase in which the hind limb was raised and the limb was not rotated but stretched outwards and retracted immediately. The duration of a foot flash was shorter than that of a foot flag and averaged 0.83±0.15 s (n = 8). Foot flashing was only observed immediately following an advertisement call.

Arm waving, upright posture and crouching were observed during close-range male-male encounters. Open-mouth displays involved elevating the head while exposing the whitish inner surface of the mouth.

One female was seen to foot flag in an aggregation of males. As in the male display the foot was rotated in an upward, backward arc exposing the whitish webbing between the toes. Within a three min period, the same female also gave several upright displays, an open mouth display, and vocalized twice. The call was a feeble, single note that could be heard by the observer, but could not be extracted from the video because of the background noise. The context in which these signals were given appears to have been intersexual. All visual signals by both males and females were dynamic visual signals that can be turned on and off by the signaller.

A “leg-snout touch” tactile display was observed between a male and a female on one occasion. After having been approached by a female, the male turned his back on her and extended his right leg toward her until his toes touched her snout. Seven seconds later the right leg was retracted and the left leg extended in the same fashion. After 12 s the procedure was repeated. The male then gave an advertisement call and jumped out of view. Further interactions between the two individuals could not be seen and it remains unclear if the male and female went into amplexus as might be expected.

### Call characteristics

**Figure 2:**
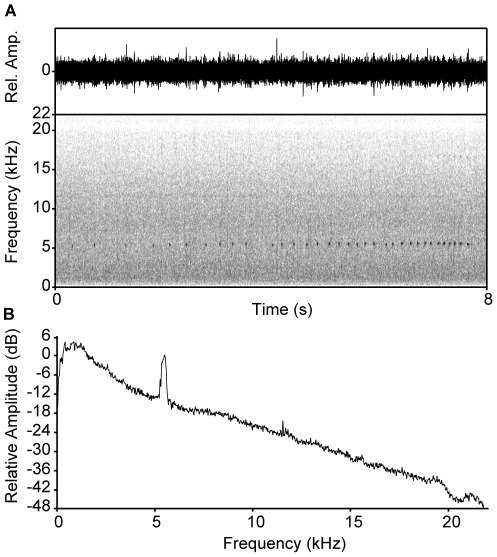
Characteristics of the advertisement call of *Staurois parvus* and its acoustic environment. (A) Oscillogram and spectrogram of a representative advertisement call with 34 notes. (B) Power spectrum of the same recording showing the energy contained in the ambient noise produced by the fast-flowing stream at which males called. The peak at 5500 Hz represents the advertisement call of *S. parvus*.

We recorded a total number of 141 advertisement calls of 14 males of *S. parvus* (all results ± SE). The energy of the call was concentrated in a narrow frequency band and consisted of on average 35±3 short pulsed notes with a dominant frequency of 5578±53 Hz (range 5295–5854 Hz; Fig. 2a). The maximum sound pressure of calls of 11 recorded individuals was 0.023 Pa±0.002 (SPL = 62 dB; range 0.001–0.126 Pa) at a distance of 1 m. The maximum sound pressure of the ambient background noise averaged 0.082 Pa±0.001 (SPL = 72 dB; n = 11) and within the call frequency 0.010 Pa±0.001 (SPL = 54 dB; n = 11; Fig. 2b). Thus, the difference in sound pressure between advertisement calls and background noise at 5578 Hz was 8 dB.

**Figure 3:**
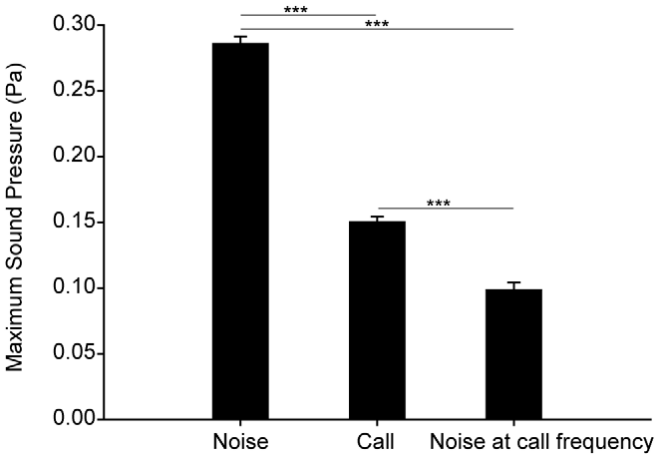
Maximum sound pressure (square-root transformed values + S.E.) of noise, advertisement call, and noise within a frequency filter in the range of the calls of 11 *Staurois parvus* males (Student's t-test: ***P<0.001).

Overall, the sound pressure between advertisement calls and noise differed significantly (LMM: *F*_2,242.8_ = 1560.732, *P*<0.001). The pairwise comparison of sound pressure between call and noise indicated that the maximum amplitude of the call had less energy than the ambient noise (call - noise: *ß* = −0.136; S.E. = 0.004; df = 195; *t* = −32.464; *P*<0.001; Fig. 3) but significantly more energy than the noise at its dominant frequency (call – noise at call frequency: *ß* = 0.052; S.E. = 0.004; df = 195; *t* = 12.409; *P*<0.001; Fig. 3). The dominant frequency of the noise was lower than the dominant advertisement call frequency (noise - call: *ß* = −5097; S.E. = 23; df = 186; *t* = −221.593; *P*<0.001).

**Figure 4:**
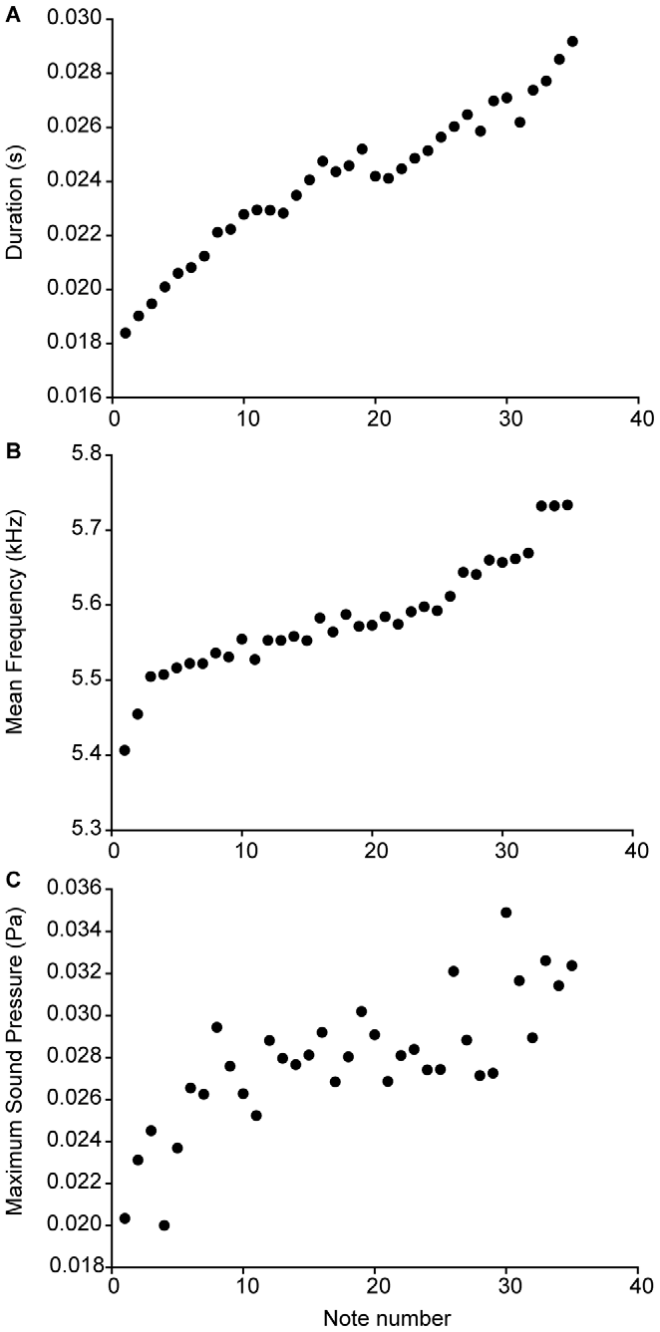
Scatterplots of the first 35 notes of the advertisement call of *Staurois parvus* of (A) mean note duration (n = 14), (B) mean frequency (n = 14) and (C) maximum sound pressure (n = 11). Plots show means of the original data (not estimates of the LMMs) for illustration that do not correspond directly with the statistical results.

Call duration (duration - note number: *ß* = 2×10^−4^; S.E. = 5×10^−6^; df = 3417; *t* = 44.452; *P*<0.001), call frequency (frequency - note number: *ß* = 6.19; S.E. = 0.421; df = 2018; *t* = 14.721; *P*<0.001) and sound pressure (sound pressure - note number: *ß* = 0.0012; S.E. = 6×10^−5^; df = 1652; *t* = 21.889; *P*<0.001) increased with note number (Fig. 4).

### Patterns of signalling activity

**Figure 5:**
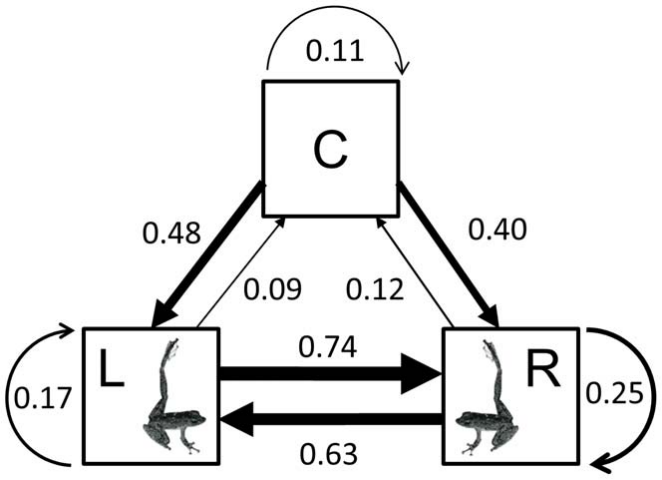
Transitional frequency matrix between three signalling behaviours (two visual and one acoustic) shown by *Staurois parvus*. C, L, and R stand for advertisement call, left foot flag and right foot flag, respectively. Width of arrows and their direction show the probability of one behaviour occurring after another behaviour was shown and the sequence of those behaviours. Numbers next to arrows designate the transitional probabilities.

In general, foot-flagging was accompanied by advertisement calling throughout all periods of the day. A representative sequence of signalling behaviours of one male over a period of 10 minutes was CRLRLCLRLRCRLRLRLCLRRRL where C denotes an advertisement call, R denotes a right foot flag, and L denotes a left foot flag. There was a high degree of association between the three behaviours (*X* ^2^_4_ = 169.6, *P*<0.01). In particular, a left foot flag was strongly associated with a right foot flag and vice versa (Fig. 5). A male giving a right foot flag will follow it with a left foot flag 63% of the time. Likewise, a left foot flag is followed with a right foot flag 74% of the time suggesting that males usually alternate between left and right foot flag. There was also a high transition probability between advertisement call and foot flag. An advertisement call was followed by a foot flag 88% (R or L: 40% or 48%) of the time while a foot flag was followed by an advertisement call only 9–12% of the time. This suggests that advertisement calls are more likely to be followed by foot flagging than foot flagging by advertisement calling. The transition probabilities also indicate that both foot flag of the same leg and advertisement call will unlikely follow itself in the behavioural sequence.

### Timing relationship between calls and foot-flags

**Figure 6:**
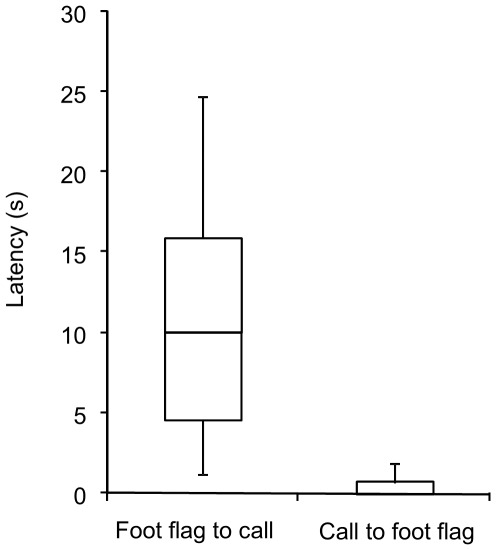
Comparison of timing relationships between advertisement call and foot flagging display of 19 *Staurois parvus* males. Box plots show the median response with interquartile range and 10^th^ and 90^th^ percentile.

The timing relationship between advertisement calls and foot flags was measured for 19 males for which at least ten observations of foot flags were available. The average delay between an advertisement call and a foot flag was 0.57±1.2 s (range 0.0–5.1 s, n = 19). In contrast, the average delay between a foot flag and a subsequent advertisement call was 11.0±7.6 s (range 1.2–24.8 s, n = 19). The time delay between advertisement call and foot flag was significantly shorter than between foot flag and advertisement call (Wilcoxon matched pairs, *Z* = 3.82, *P*≤0.001, n = 19; Fig. 6).

### Acoustic playback experiments

**Figure 7:**
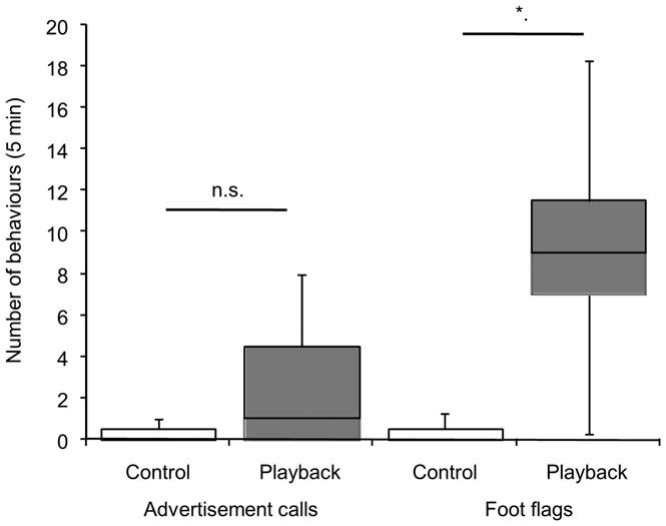
Responses of seven male *Staurois parvus* to silent control (pre-playback) and playback of synthetic advertisement calls. Box plots show the median response with interquartile range and 10^th^ and 90^th^ percentile. *P<0.05, n.s. = non-significant.

Variation in sound pressure level of the playback had no significant effect on the number of advertisement calls or foot flags given by males (least squares linear regression, r^2^ = 0.03, n.s. and r^2^ = 0.07, n.s., respectively). Males produced both advertisement calls and foot flags in response to synthetic advertisement calls. Significantly more foot flags (9.25±6.8) were given during the playback period then during the pre-playback period (Wilcoxon matched pairs, *Z* = 2.20, *P*<0.05, n = 7; Fig. 6). Although an increase was also shown in the number of advertisement calls given in response to the playback, this increase was not significant (Wilcoxon matched pairs, *Z* = 1.83, n.s., n = 7; Fig. 7). During the playback period, males produced significantly more foot flags than calls (Wilcoxon matched pairs, *Z* = 2.37, *P*<0.05, n = 7).

## Discussion

This study reinforces the findings that acoustic and visual displays are functionally linked in the genus *Staurois*. Grafe & Wanger [25] documented that the advertisement calls and foot flags of *S. guttatus* form a functional unit as a multicomponent and multimodal display. Their results suggested that the advertisement calls have an alerting function by drawing the attention of the receiver to the subsequent dynamic foot flag. Likewise, *S. latopalmatus* males use short calls in conjunction with foot flags for intra- and interspecific communication with short calls preceding foot flags [22] and *S. tuberilinguis* often give foot flags right after calling [36].

Males of *S. parvus* used advertisement calls and foot flags closely combined throughout the day under varying light conditions. The timing relationship between the acoustic and visual signal supports the alerting signal hypothesis as an explanation for multimodal communication [28]. The latency between foot flags and calls was significantly higher than between calls and foot flags. In addition, the playback experiments suggest that one function of the advertisement call is to alert receivers to the subsequent visual foot flag. The acoustic playback elicited both acoustic and visual signalling not just advertisement calling or foot flagging as would be expected if acoustic and visual signals were not linked. Furthermore, males gave significantly more foot flags than calls during advertisement call playback suggesting that the visual display may be the more informative signal with calls used predominantly to gain a receivers attention.

In addition to foot flags, male and female *S. parvus* show numerous, less frequently observed visual displays that need to be explored further. Similar to foot flagging, the much faster foot flashing was also seen to be closely synchronized with advertisement calling suggesting a similar function in territorial intra- and intersexual signalling, but possibly given when males are more excited when approached by a female. The other visual and tactile signals appear to be used for close range communication. In particular, the leg-snout touch tactile display between a male and female, not previously reported in the genus *Staurois*, suggests that mate choice occurs after females approach a male. Similar tactile displays have been shown to occur in *Hyla ehrhardti*, a frog that also uses foot flagging as a visual display and in which males lead females to oviposition sites [37].

The visual display in *S. parvus* typically closely follows acoustic signalling. In contrast, multimodal signals in many other anurans are often simultaneous displays given most notably when the vocal sacs are inflated during calling [38]–[41]. Foot flagging allows for more flexibility as the visual and acoustic signals can be uncoupled and used to different degrees as the ecological and social environments change.

As in *S. gutattus*, background noise may be necessary but not sufficient in explaining foot-flagging in *S. parvus* because such noise has not led to foot-flagging behaviour in other anurans that call at night near running water in the same habitat [25]. An additional correlate of visual signalling appears to be diurnality albeit with exceptions [26].

Our results indicate that vocal communication in *S. parvus* is not impaired by abiotic background noise. The high-frequency advertisement call does not overlap with dominant frequencies of the stream. Two major evolutionary trajectories seem to have been followed by male anurans in their need to avoid broadband low-frequency-dominated masking noise. First, to increase call dominant frequency above the background noise [14],[21],[42]. Such spectral shifts have been documented most notably in *Odorrana tormota* and *Huia cavitympanum* in which males call in the ultrasonic range [14],[42]. However, morphological constraints of body size and the inherent transmission limitations caused by the high rate of attenuation and degradation of high frequency sounds may limit widespread use of this solution. Secondly, males that switch to the use of visual signals as the prime mode of communication will be at an advantage, since continuous, chronic noise found along fast flowing streams will favour the evolution of signalling in modalities less affected by noise [15].

Correlations of body size and call frequency of ranid frogs indicate that all investigated species of the genus *Staurois* display calls with higher frequencies than expected from their body size [21]. These shifts in signal frequency clearly facilitate communication in the presence of high-intensity background noise as observed in this study. Likewise, other frog and bird species are able to increase the pitch of their calls or songs while vocalizing in areas of high ambient noise [12],[13],[43]–[45].

Additional features of the advertisement call of *S. parvus* facilitate communication under continuous background noise and distinguish it from *S. guttatus*. *Staurois parvus* produces an advertisement call that varies in note number, ranging from 23 to 54 notes per call. We observed a continuous increase in frequency, sound pressure and duration with increasing note number. We interpret the production of very repetitive notes as a redundant feature of the calling behaviour of *S. parvus* that facilitates communication by enhancing the contrast with high levels of continuous background noise. Increased song duration, and/or increased call or note rate has been shown to be a response by a wide range of animals to increases in background noise [9],[46]–[48], and females are known to prefer calls with greater intensity, higher call rate and duration [49],[50]. The additional increase in sound pressure and note duration with increasing note number could be interpreted as an attempt to increase signal range in a graded manner. Instead of producing a short long-range signal, males produce longer calls that increase communication range with increasing note number. Thus, receivers at close range will be targeted immediately while those further away will be reached only with later notes, presumably saving energy. This suggests that males can adjust note number depending on proximity of receivers and background noise levels and resembles that of graded aggressive calling in other anurans [51]. Finally, the energy of the advertisement call of *S. parvus* is also concentrated in a narrow frequency band. Such narrowly tuned calls presumably facilitate communication in noisy environments [52],[53].

Several studies have demonstrated that chorus noise produced by conspecifics and anthropogenic noise can interfere with female call detection and perception [44],[54],[55]. A threshold for detection of at least +1.5–3.0 dB seems to be critical for females to be able to detect males. *Staurois parvus* males generally produce no overlapping calls or choruses but communicate during constant background noise. A sound pressure difference of 8 dB, as shown in this study, should be a more than sufficient threshold for female detection. Female anurans have been shown to discriminate sounds even under lower signal-to-noise ratios [56]–[58]. In our study area, females are rarely seen with the exception of pairs in amplexus and thus their phonotactic behaviour remains unknown.

It should be noted that *S. parvus* males communicate in an environment of near continuous noise created by running water and thus solutions used by other animals to communicate in environments with fluctuating noise levels may not be appropriate. Noise generated by social aggregations usually fluctuates in time and thus receivers may adapt by evolving mechanisms that exploit such fluctuations [10],[59]. Release from masking can occur by receivers listening in the gaps or dips of fluctuating noise, a solution to the cocktail party effect encountered by human listeners [60],[61]. In addition, spatial release from masking [19] is difficult to achieve because males call in close proximity to running water from the stone surface of the waterfalls. Thus, both gap or dip listening and spatial release from masking may not be viable alternatives for receivers, increasing the selective pressure on male *S. parvus* to use visual signals to communicate.

Although background noise in the environment of *S. parvus* is nearly continuous over a time period of minutes to hours, it will vary strongly depending on rainfall. Especially in smaller streams with small catchment areas that are typical habitats of *S. parvus*, background noise levels will vary considerably between days and between dry and wet seasons. Multimodal signalling will be favoured under such fluctuating ecological environments if each modality is favoured under different conditions. Acoustic signalling will be at an advantage under more quiet conditions and low light levels, whereas visual signals will prevail when the noise of rushing water is high and light levels provide the best contrast. Such context-dependent dynamic selection regimes are recently gaining wider attention [62],[63] and enhance our understanding of the flexibility seen in the use of multimodal signals in *S. parvus*.

We conclude that *S. parvus* has solved the problem of continuous broadband low-frequency noise by modifying the amplitude, pitch, repetition rate and duration of notes within their advertisement call in addition to using numerous visual signals, foot-flagging being the most conspicuous. Such a multi-pronged approach has not been documented before in amphibians. It seems likely that background noise has driven the evolution of multimodal communication. Indeed, foot-flagging has evolved independently mainly in anuran species that communicate along fast-flowing streams [15]. Playback experiments using visual foot-flagging signals would be particularly useful to further our understanding of the communication system of frogs in the genus *Staurois*.
